# Determining minimal clinically important differences in ecological momentary assessment measures of fatigue in people with multiple sclerosis

**DOI:** 10.1007/s11136-025-03948-5

**Published:** 2025-03-22

**Authors:** Sonia Sharma, Tiffany J. Braley, Kevin N. Alschuler, Dawn M. Ehde, Anna L. Kratz

**Affiliations:** 1 Department of Physical Medicine & Rehabilitation, University of Michigan, Michigan Medicine, Ann Arbor, MI USA; 2 Department of Neurology, Division of Multiple Sclerosis and Clinical Neuroimmunology, University of Michigan, Michigan Medicine, Ann Arbor, MI USA; 3 Department of Rehabilitation Medicine, University of Washington School of Medicine, Seattle, WA USA; 4 Department of Medicine, Division of Behavioral Medicine, Jacobs School of Medicine and Biomedical Science, University at Buffalo, Erie County Medical Center, 462 Grider Street, Buffalo, NY 14215 USA; 5 Department of Neurology, University of Washington School of Medicine, Seattle, WA USA

**Keywords:** Minimal clinically important differences, Ecological momentary assessment, Multiple sclerosis, Fatigue

## Abstract

**Purpose:**

Fatigue is a common debilitating symptom of multiple sclerosis (MS). Ecological momentary assessment (EMA) provides a more reliable and sensitive assessment of fatigue outcomes relative to traditional recall surveys; however, the minimal clinically important difference (MCID) for EMA fatigue outcomes has not been established.

**Methods:**

MCIDs for EMA fatigue intensity and fatigue interference (0–10 numerical rating scale) that were assessed as outcomes in a pragmatic randomized clinical trial of three fatigue interventions were determined using two statistical approaches. The Patient Global Impression of Change (PGIC) and the Modified Fatigue Impact Scale (MFIS) were used within the anchor-based approach, and standard deviations (SD) and standard error of measurements (SEM) were examined within the distribution-based approach.

**Results:**

Pre- and post-treatment EMA data from 336 individuals with MS (76.2% female, 71.1% relapsing–remitting MS, mean age 48.8 (± 11.7) years, mean duration of MS 12.2 (± 9.8) years) were included in the analysis. Percent complete EMA data (4 EMAs/day) for 7 days were comparable pre- and post-treatment for fatigue intensity and for fatigue interference. Using the PGIC and MFIS anchors, change in EMA scores averaged 0.94 and 1.04 for fatigue intensity and 0.62 and 1.04 for fatigue interference, respectively. The SD and SEM for EMA fatigue intensity were 0.75 and 1.19 and for EMA fatigue interference were 0.83 and 1.30, respectively.

**Conclusion:**

Combining two approaches, our study contributes foundational information regarding meaningful change on EMA measures of fatigue, enabling effective use of EMA to assess fatigue treatment outcomes in a person-centered manner.

## Plain Language Summary

The smallest changes in treatment outcomes considered important by patients are often determined for traditional recall surveys. However, recall-bias with traditional surveys may contribute to unreliable assessment of the outcome. Evidence suggests that ecological momentary assessments (EMA; real-time self-report) can provide a more reliable and sensitive assessment of symptoms relative to traditional surveys. Applying two analytic methods to investigate meaningful changes of EMA fatigue outcomes involving patient-oriented and sample specific measures of variability, our work addresses the challenge of recall-bias affecting assessments with traditional surveys and provides a practical way to assess meaningful change in fatigue treatment outcomes.

## Introduction

Multiple Sclerosis (MS) is an autoimmune disease of the central nervous system that affects approximately 2.5 million people worldwide [1]. Although MS is classically associated with motor impairment and gait disturbances, individuals with MS typically experience a constellation of physical and cognitive symptoms including fatigue that impact health-related quality of life [2–15]. Self-reported fatigue, defined as “a subjective lack of physical or mental energy that is perceived by the individual or caregiver to interfere with usual and desired activities” [16] is a pervasive symptom in MS. Fatigue, often associated with greater activity restrictions, pain severity, sleep disturbances, depressive symptoms, cognitive problems, physical, and functional disability [17, 18], indirectly accounts for over 80% of the variance in health-related quality of life (HRQoL) in people with MS (pwMS) [19]. These findings suggest that to improve HRQoL in pwMS, management of self-reported fatigue is imperative. However, recent findings show that self-reported fatigue, like other chronic self-reported symptoms in MS, is not static, even over a short time frame within a day [20–24].Among common MS symptoms, fatigue was the most highly variable symptom across and within days [23]. This variability can make it difficult for people to rate their fatigue severity on traditional self-report measures that require recall and summation of fatigue over days to weeks [24]. Even in the absence of very high variability, recall bias, which trends toward more recent and extreme symptom experiences, can contribute to an unreliable assessment of the construct of interest [25].

Ecological momentary assessments (EMA) capture self-report of symptoms in real time, as they are experienced in daily life [26, 27]. Compared to traditional recall measures, EMA is shown to provide a more reliable and sensitive assessment of symptoms [28, 29]. While concerns of over-medicalization from use of EMA in MS care have been raised [30], the dynamic view provided by linking MS symptoms to environmental and contextual factors through EMA helps identify how daily activities and health-related quality of life are impacted by these clinical manifestations [31].

Advances in technology (e.g. wearables, smartphone apps) and EMA methodology over recent decades have significantly increased the use of EMA in health research. Despite the increased use of EMA methods and their advantages, few clinical trials have employed EMA to measure study outcomes. One reason for this may be the lack of established cut-points for clinically significant change for constructs measured with EMA. For example, minimal clinical important difference (MCID) is often used to interpret the minimal threshold for magnitude of change when comparing treatments, groups of patients, or within one group over time [32]. MCID is an essential statistical consideration in trial design [33]. However, MCIDs for important EMA fatigue outcomes – self-reported fatigue intensity defined as the degree or severity of tiredness or exhaustion a person experiences (“What is your level of physical (bodily) fatigue right now?” and “What is your level of mental (brain) fatigue right now?”) and self-reported fatigue interference defined as the extent to which feelings of tiredness or exhaustion negatively impact a person's ability to perform their usual daily activities (“How much is physical fatigue interfering with what you are doing right now?” and “How much is your mental fatigue interfering with what you are doing right now?”) have not been established for use in MS or in other chronic health conditions. To address this gap, this study leveraged data from a randomized clinical trial for MS fatigue to determine the reliability of the EMA fatigue measures and the EMA based MCID scores for fatigue intensity and fatigue interference.

## Material and methods

### Study sample and procedures.

This study reports on EMA measures of fatigue outcomes from a multisite, randomized pragmatic trial, conducted among participants with MS (NCT03621761). The trial protocol was approved by the Institutional Review Boards of the University of Michigan Medical School and University of Washington before initiation of study activities and has been published elsewhere [34, 35].

In brief, N = 336 adults with MS and chronic fatigue were recruited through two academic MS Centers and surrounding neurology practices. Participants age ≥ 18 years, with a neurologist-confirmed MS diagnosis, and chronic, problematic fatigue that in the opinion of the patient, has interfered with their daily activities for ≥ 3 months were included. Recent MS relapse, current use of wake-promoting agent, hormonal contraception, pregnancy, breastfeedling, hypersensitivity to modafinil and medical conditions that could affect participant safety or eligibility were considered exclusionary [35]. Participants were randomized 1:1:1 to receive 12-weeks of modafinil (a wake-promoting agent), one-on-one telephone delivered cognitive behavioral therapy (CBT) for fatigue, or a combination of both modafinil and CBT. The primary outcome was change in fatigue impact from baseline to 12-weeks (primary endpoint), as measured by the Modified Fatigue Impact Scale (MFIS). EMA measures of fatigue intensity and fatigue interference (secondary outcome measures) were collected for 7 consecutive days at baseline (before randomization) and during the last week of treatment prior to treatment discontinuation. EMAs were collected via the PRO-Diary (CamNTech, Cambridge, UK), a user-interface enhanced wrist-worn accelerometer for collection of real-time self-reported data. Participants logged their symptoms rating four times per day. Ratings at wake time and bedtime were self-initiated. Two quasi-random midday assessments between 11 am and 7 pm (i.e. random prompts within two windows: 11AM-3PM, 3PM-7PM) were prompted by an audible alert [35].

### Measures used to determine MCID scores

Momentary fatigue intensity was assessed with two items: “What is your level of physical (bodily) fatigue right now?” and “What is your level of mental (brain) fatigue right now?”.

Momentary fatigue interference was assessed with two items: “How much is your physical fatigue interfering with what you are doing right now?” and “How much is your mental fatigue interfering with what you are doing right now?” Items were rated on a numerical rating scale (NRS) of 0 (no fatigue) to 10 (extremely severe fatigue) for fatigue intensity and 0 (no interference) to 10 (totally interfering; not able to do what I want because of fatigue) for fatigue interference. The measures of mental and physical EMA fatigue intensity and EMA fatigue interference, generally considered as state periods, were created to enhance reliability beyond a single item alone and were based on the NRS of fatigue that have been used before in MS research [17, 23, 36, 37].Composite measures were created in recognition of other validated fatigue questionnaires [38, 39] where either items or smaller factors on mental and physical fatigue were incorporated. The average score of the mental and physical fatigue intensity responses and the average score of the mental and physical fatigue interference responses were computed with higher scores indicating higher fatigue intensity or higher fatigue interference. Pre- and post-treatment assessment scores for fatigue intensity and fatigue interference were then created by averaging across all the EMA ratings at baseline (pre-treatment) and at 12-weeks (post-treatment), each considered as measures of trait fatigue over a short 7-day period of time. These aggregated values were used to then calculate the mean pre-to-post-treatment change scores for EMA fatigue intensity and fatigue interference.

The Patient Global Impact of Change (PGIC) is a 7-point self-report measure designed to assess patient's perception of overall improvement following treatment in clinical trials and was administer post-treatment at 12-weeks [40]. Participants rated their change from 1 = “no change (or condition has got worse)”, to 7 = “A great deal better, and a considerable improvement that has made all the difference”. A threshold-change of 5 = “moderately better, and a slight but noticeable change” on the PGIC was used as the cutoff. The within-person mean change in EMA fatigue scores at the chosen PGIC threshold of 5 was compared to the within-person mean change in EMA fatigue scores of those who reported 2 = “almost the same, hardly any change at all” on the PGIC. The difference in the mean EMA fatigue change scores between the two groups was used to calculate the between-person mean change score for EMA fatigue.

The MFIS is a 21-item self-reported measure that contains three sub-scales of physical, cognitive, and psychological impact of fatigue in people with MS [39]. The MFIS total score is calculated by summing responses on a 0–4 scale, with a minimum possible score = 0 and a maximum possible score = 84. Higher scores indicate a greater impact of fatigue. Psychometric properties show excellent reliability with Cronbach α values ranging from 0.81 to 0.95 for total and sub-scales [41]. A change score of ≥ 10 points on the MFIS is considered clinically relevant [42]. The mean change in EMA fatigue scores at the ≥ 10 point MFIS threshold was used to determine the MCID for within-person mean change scores and was compared to the mean change in EMA fatigue scores of those who reported < 10 point change on the MFIS. The difference in the mean EMA fatigue change scores in those with < 10 point vs ≥ 10 point change on the MFIS was used to determine the between-person mean change score for EMA fatigue.

### Methods and statistical analysis

Anchor-based and distribution-based approaches were used to derive EMA based fatigue intensity and interference MCID scores. We also combined on these approaches to consolidate MCID estimates and systematically determine the most appropriate value for the EMA-based MCID for fatigue intensity and fatigue interference.

#### Anchor-based approach

In this approach, change in EMA assessed mean fatigue intensity and mean fatigue interference were compared to the PGIC, and the MFIS, referred to here as “anchors”. Alternatively, the change in mean EMA fatigue intensity and interference scores was conceptualized as a diagnostic test for detecting individuals who have experienced clinically important improvement in fatigue, that is, reporting at least the threshold or greater improvement on the anchor questions which served as the reference standards. With the use of receiver operating characteristic (ROC) analysis, the MCID of EMA fatigue was calculated using a threshold change of ≥ 5 i.e. moderately better or more vs ≤ 4 i.e. somewhat better or less on the PGIC, and a ≥ 10-point vs < 10-point improvement between pre-treatment baseline and post-treatment 12-weeks MFIS scores. The proportion of agreement between the EMA fatigue measures and the reference measures of PGIC and MFIS yielded accuracy statistics similar to diagnostic procedures for classification analyses.

#### Distribution-based approach

In this approach the MCIDs were referenced to a measure of variability or effect size of the EMA fatigue outcomes. Values ranged from the smallest amount of change, that is standard error of measurement (SEM), to very large change, that is pre-treatment baseline standard deviation (SD). To determine the distribution-based values across the 7 days, reliability estimates of the repeated momentary fatigue assessments were calculated across the 7-day assessment period. We assessed the average between-person reliability and the intraclass correlation (ICC), the expected correlation between two randomly sampled measurements from the same person, by aggregating across the 7-day assessment period. In addition, effect sizes were used to determine, on the average, the between subject comparison of improvements in EMA fatigue relative to the degree of variability and were also estimated by aggregating across the 7-day period. Cohen’s d classification of 0.2 – < 0.5 small; 0.5 – < 0.8 moderate; ≥ 0.8 large was used to determine the strongest effect across the various distribution-based indicators.

Next, the distribution-based indicators were combined with the anchor-based measures to systematically determine the most appropriate value for the MCID. In this approach, PGIC and MFIS at their respective thresholds of 5 vs ≤ 4 and ≥ 10-points vs < 10-points were used as the reference measures against which accuracy, sensitivity, specificity, and the Youden’s Index for each of the EMA fatigue distribution-based indicators were determined. MCID values that corresponded to the highest accuracy and Youden’s Index were identified.

Analyses were performed using SAS (version 9.4). Descriptive statistics were generated for sociodemographic characteristics. Correlations were computed using Spearman and Pearson correlations between the mean change in EMA fatigue scores and PGIC (ordinal) and MFIS (interval), respectively. Mean change in EMA fatigue scores were calculated for each of the 7 ratings of improvement on the PGIC and at ≥ 10-point vs < 10-point change on the MFIS. ROC curve estimates of area under the curve (AUC), sensitivity, specificity, accuracy, Youden’s Index, and the Euclidean distance were calculated. To evaluate reliability, unconditional means multilevel models were generated for fatigue intensity and fatigue interference. These models were run using fatigue scores from assessment periods of increasing duration (e.g. day 1 alone, days 1–2, days 1–3, etc.) and estimated the number of days of assessment that obtain between-person reliabilities of > 0.70, > 0.80, and > 0.90. Between-person reliabilities for fatigue intensity and fatigue interference were calculated using formulas provided by Raykov & Marcoulides [43], whereby the average number of completed session were calculated as total number of test sessions completed/total number of possible test sessions during the respective time duration. The between-person and within-person variances were used to calculate intraclass correlations (ICC), which was used as the measure of reliability to calculate the SEM. The minimal detectable change (MDC) and standard error of measure of change (SEMc) were computed using formulas from Maassen et al. [44]. All main analyses were performed collapsing treatment arms. Differences in PGIC ratings by treatment arm and demographic characteristics were explored and the within- and between-person mean change in EMA fatigue scores across PGIC categories 2 and 5 were computed to determine degree of change in MCID by these groups. Differences in demographic characteristics between individuals who provided EMA fatigue ratings pre- and post-treatment and those who had missing data at either time points were also determined to address potential bias due to attrition.

## Results

### Participant and EMA descriptives

Participants were on average 48.8 (± 11.7) years old, predominantly female (76.2%), White (85.37%), non-Hispanic or Latino (90.77%), unemployed (47.32%), married (58.04%), and had a college graduate level education (38.69). The average duration of MS was 12.2 (± 9.8) years with relapsing–remitting MS as the predominant subtype (71.1%), Table 1.

**Table 1:** Participant demographic characteristics

Participants, n	N = 336
Age (years), mean ± SD (range)	48.81 ± 11.65 (57.0)
Time since diagnosis (years), mean ± SD (range)	12.16 ± 9.81 (0–62.0)
MS type, % (n)	
Relapsing Remitting MS	71.13 (239)
Secondary Progressive MS	19.35 (65)
Primary Progressive MS	8.63 (29)
Missing	0.89 (3)
Self-reported Sex, % (n)	
Female	76.19 (256)
Male	23.81 (80)
Work status, % (n)	
Full time	37.20 (125)
Part time	11.90 (40)
Seasonal	0.30 (1)
Unemployed	47.32 (159)
Unknown	2.08 (7)
Missing	1.19 (4)
Highest level of education, % (n)	
9th grade or less	0.30 (1)
High school graduate or GED	5.36 (18)
Vocational or Technical School	3.87 (13)
Some college	25.60 (86)
College graduate	38.69 (130)
Graduate school or professional school	25.60 (86)
Missing	0.60 (2)
Race, % (n)	
White	85.37 (286)
African American or Black	7.16 (24)
Asian	1.19 (4)
American Indian or Alaska Native	0.30 (1)
Native Hawaiian or Other Pacific Islander	0.60 (2)
Other	1.79 (6)
Mixed	3.58 (12)
Missing	0.30 (1)
Ethnicity, % (n)	
Hispanic or Latino	5.36 (18)
Not Hispanic or Latino	90.77 (305)
Unknown	2.38 (8)
Marital status, % (n)	
Divorced	18.15 (61)
Domestic partner	3.87 (13)
Legally separated	0.89 (3)
Married	58.04 (195)
Never married	14.58 (49)
Widowed	3.87 (13)
Unknown	0.30 (1)
Missing	0.30 (1)

Of the 336 people with MS, across the 7-day assessment period, 324 individuals provided ≥ 1 fatigue ratings per day at baseline and 281 individuals provided ≥ 1 fatigue rating per day at 12-weeks. The average percent complete EMA data (4 EMAs/day) for 7 days were comparable pre- and post-treatment for fatigue intensity and fatigue interference. Across the 7-day assessment period, on average, 79.99% (1036.67/1296) of EMA fatigue intensity ratings at baseline (mean (sd) = 4.66 (1.40)) and 69.78% (784.33/1124) at 12-weeks (mean (sd) = 3.69 (1.48)) were completed per day, and 79.77% (1033.83/1296); for EMA fatigue interference ratings at baseline (mean (sd) = 3.63 (1.44)) and 69.53% (781.50/1124) at 12-weeks (mean (sd) = 2.72 (1.62)) were completed per day. On comparing demographic characteristics of individuals with missing EMA fatigue change score ratings to those for whom a change score could be computed, individuals with missing EMA fatigue were slightly younger (mean (sd) = 46.11 (11.26); p-value = 0.04) compared to those with non-missing EMA fatigue change scores (mean(sd) = 49.43 (11.68)). Correlations between the MFIS and average EMA fatigue intensity ratings at baseline (r = 0.52) and at 12-weeks (r = 0.60), and between the MFIS and average EMA fatigue interference ratings at baseline (r = 0.53) and at 12-weeks (0.60) were all significant (all p < 0.001).

### Correlation between EMA scores and anchor-based measures

Correlations between each of the anchor-based measures (PGIC ratings and MFIS mean change scores) and the EMA fatigue mean change scores were ≥ 0.35 (all p < 0.001), Table 2.

**Table 2:** Correlation between Anchors and Mean Change Scores in Fatigue Intensity and Interference

Anchors x mean change scores in EMA fatigue	r	p-value
PGIC ratings x fatigue intensity	− 0.37	< 0.001
PGIC ratings x fatigue interference	− 0.36	< 0.001
MFIS mean change scores x fatigue intensity	0.42	< 0.001
MFIS mean change scores x fatigue interference	0.41	< 0.001

Anchors: Patient Global Impression of Change (PGIC) and Modified Fatigue Impact Scale (MFIS)

Mean change scores: mean difference between pre- and post-treatment score

*r* Pearson’s or Spearman’s correlation coefficient; p-value at alpha = 0.05

### Anchor-based scores

**Fig. 1:**
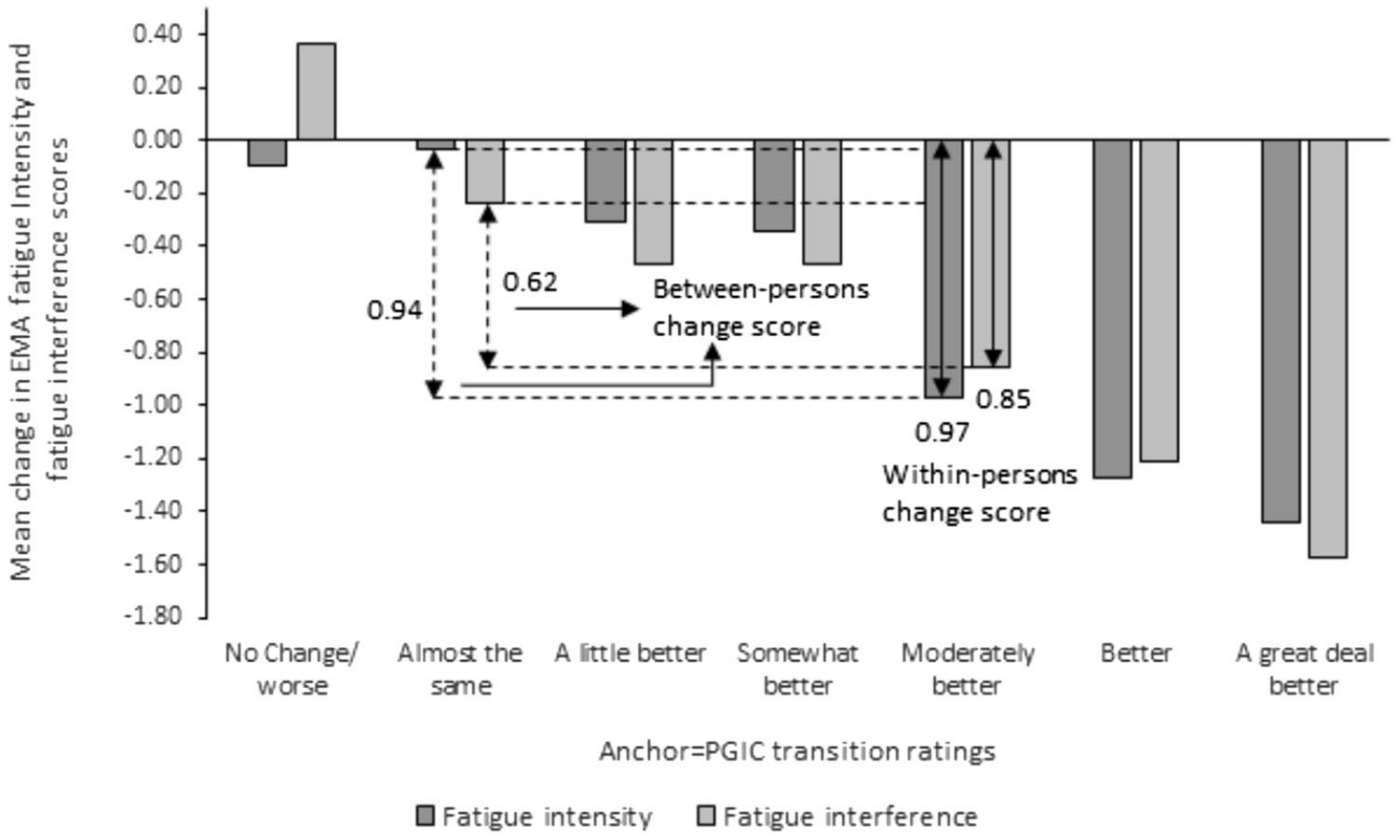
Mean change in EMA fatigue intensity and fatigue interference scores for people with MS is plotted against PGIC ratings. The minimal clinically important difference (MCID) calculation using just the mean change in EMA fatigue scores for individuals reporting “moderately better” on the PGIC is shown with the solid double arrow and depicts the within-person mean change score. The MCID calculation using the mean change in EMA fatigue scores for individuals reporting “moderately better” on the PGIC and subtracting the mean change in EMA fatigue scores for the “almost the same” transition rating is shown with the dotted double arrow and depicts the between-person change score

**Fig. 2:**
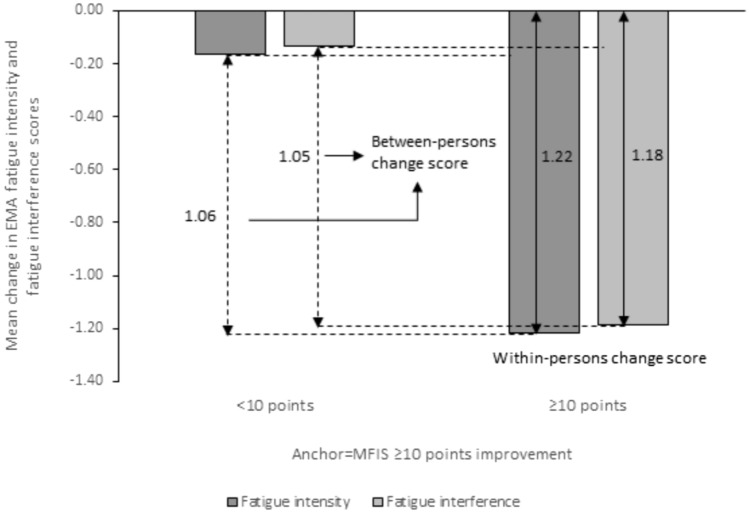
Mean change in EMA fatigue intensity and fatigue interference scores for people with MS is plotted against MFIS cut-off. The minimal clinically important difference (MCID) calculation using just the mean change in EMA fatigue scores for individuals reporting ≥ 10 point improvement on the MFIS is shown with the solid double arrow and depicts the within-person mean change score. The MCID calculation using the mean change in EMA fatigue scores for individuals reporting ≥ 10 point change on the MFIS and subtracting the mean change in EMA fatigue scores for the < 10 point improvement on the MFIS shown with the dotted double arrow and depicts the between-person change score

Mean change in EMA fatigue intensity and fatigue interference scores approximated 1.0 when anchored on PGIC and MFIS. When anchored on PGIC, the within- and between-persons change scores in mean EMA fatigue intensity (within-person = 0.97; between-persons = 0.94) and fatigue interference scores (within-person = 0.85; between-persons = 0.62) ranged from 0.62 to 0.97 (Fig. 1). When anchored on the MFIS the within- and between-person change in mean EMA fatigue intensity (within-person = 1.22; between-persons = 1.06) and fatigue interference (within-person = 1.18; between-persons = 1.05) scores were slightly above 1.0 and ranged from 1.05 to 1.22 (Fig. 2). Effective size estimates for the within-person change scores for fatigue intensity = 0.69 and fatigue interference = 0.59 using the PGIC anchor and for fatigue intensity = 0.80 and fatigue interference = 0.77 using the MFIS as the anchor were moderate to strong.

Differences in PGIC (groups 2 and 5) by treatment arm and demographic characteristics were tested and no significant differences were found (all P-value > 0.05). MCID values for the between-person difference in the EMA mean change scores fall within the range of 0.30–1.51 for EMA fatigue intensity and between 0.19 and 0.80 for EMA fatigue interference, Table 3.

**Table 3:** Within- and between-person mean change in EMA fatigue scores by treatment arm and demographic characteristics

Characteristics (n)	Mean change in EMA scores					
	Fatigue intensity			Fatigue interference		
	Within-person^a^	Within-person^b^	Between-person	Within-person^a^	Within-person^b^	Between-person
Treatment arm						
CBT (114)	0.00	− 0.89	− 0.89	− 0.51	− 0.88	− 0.37
Combo (108)	− 0.04	− 0.80	− 0.76	− 0.09	− 0.79	− 0.70
Drug (114)	− 0.05	− 1.19	− 1.13	− 0.08	− 0.88	− 0.80
Sex						
Females (256)	0.02	− 1.05	− 1.07	− 0.27	− 0.92	− 0.65
Males (80)	− 0.14	− 0.45	− 0.30	− 0.14	− 0.43	− 0.29
Race						
White (286)	0.01	− 1.04	− 1.05	− 0.24	− 0.91	− 0.66
Other (49)	− 0.39	− 0.31	0.08	− 0.15	− 0.34	− 0.19
Education						
Some college or less (118)	− 0.07	− 1.08	− 1.01	− 0.22	− 0.98	− 0.76
College graduate or higher (216)	− 0.01	− 0.93	− 0.93	− 0.24	− 0.81	− 0.57
Age						
Younger: < 49yrs (169)	− 0.47	− 0.86	− 0.39	− 0.52	− 0.99	− 0.47
Older: ≥ 49yrs (167)	0.41	− 1.10	− 1.51	0.05	− 0.69	− 0.74

EMA change scores computed using PGIC as anchor

Within-person change score computed for ^a^2 = almost the same and ^b^5 = moderately better on the PGIC

Between-person change score computed as the difference in the within-person change score from 2 = almost the same

*CBT* Cognitive Behavioral Therapy, *COMBO* Combination of CBT and Drug

**Fig. 3:**
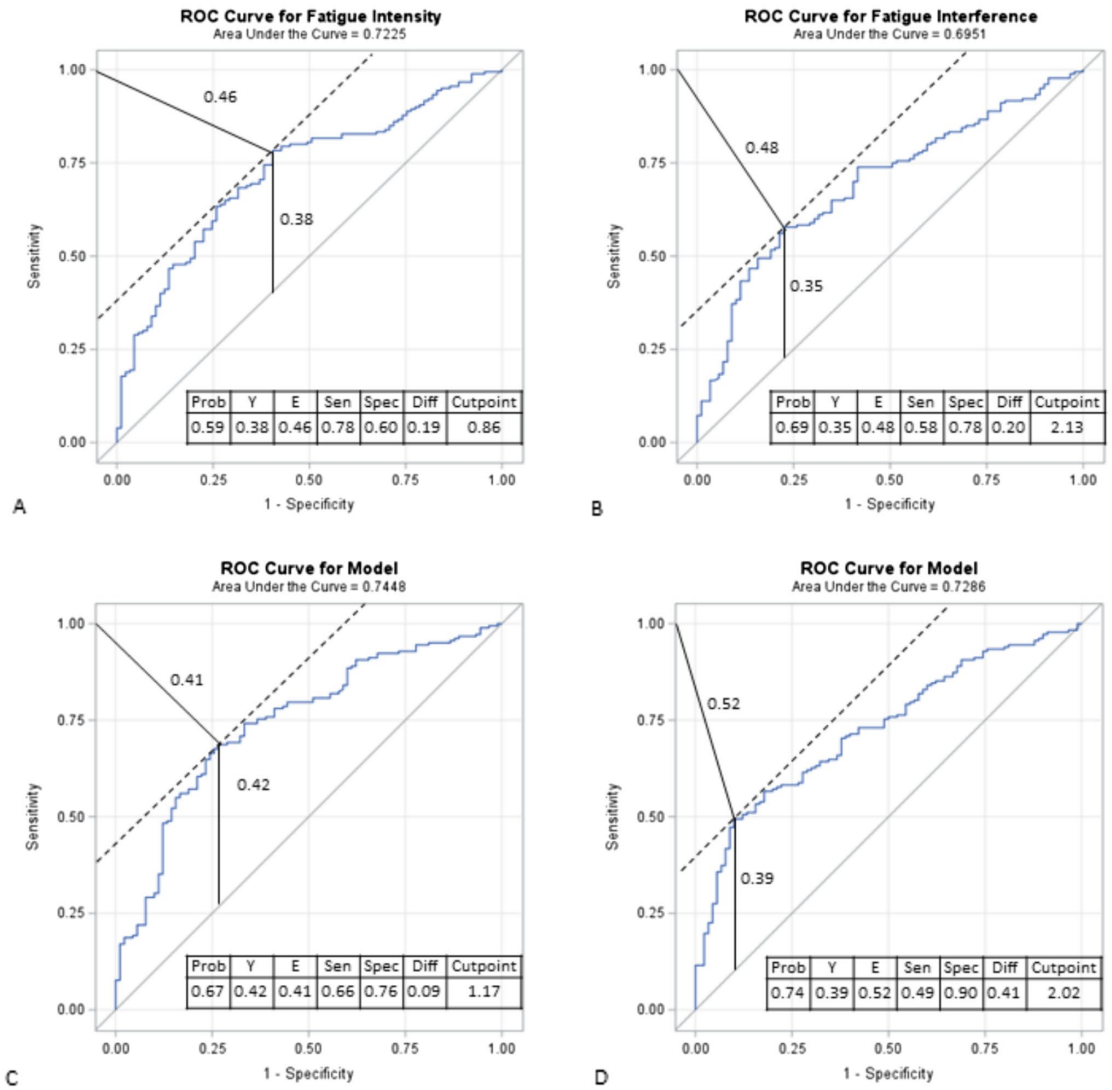
ROC curves for EMA fatigue intensity and fatigue interference illustrating Youden’s index and the Euclidean distance to determine cut-offs using anchors PGIC (**A** and **B**) and MFIS (**C** and **D**). *AUC* Area under the curve, *Sen* Sensitivity = true positives/ (true positives + false negatives); *Spec* Specificity = true negatives/ (true negatives + false positives); *Diff* Difference = Abs (sensitivity–specificity)] E: Euclidean distance = √1 − sensitivity)2 + (1 – specificity)2. The point on the ROC curve closest to (0,1) corner is used as a criterion for selecting the optimal cut-point as the point minimizing the Euclidean distance between the ROC curve and the (0,1) point; Y: Youden's Index = Sensitivity + Specificity—1.00. The point on the ROC curve that is farthest from the random chance diagonal is used as a criterion for selecting the optimal cut-point as the point maximizing the Youden's Index. Youden’s index value ranges from − 1 through 1, 0 indicates the proportion of true positives and true negatives are equal, i.e. the test is useless. 1 indicates that there are no false positives or false negatives, i.e. the test is perfect. The index gives equal weight to false positive and false negative values, so all tests with the same value of the index give the same proportion of total misclassified results

Examples of the ROC curves for EMA fatigue intensity and fatigue interference with the two anchor-based measures are shown in Fig. 3. Overall, the accuracy of the EMA fatigue measures as a diagnostic test were acceptable (all AUC≈7.0) and comparable when compared against reference measures of PGIC (AUC: fatigue intensity = 0.72; fatigue interference = 0.70) and MFIS (AUC: fatigue intensity = 0.74; fatigue interference = 0.73). The diagnostic test characteristics values for the EMA fatigue measures were also comparable when compared against the PGIC (Youden’s indices: fatigue intensity = 0.38; fatigue interference = 0.35 and Euclidean distances: fatigue intensity = 0.46; fatigue interference = 0.48) and the MFIS (Youden’s indices: fatigue intensity = 0.42; fatigue interference = 0.39 and Euclidean distances: fatigue intensity = 0.41; fatigue interference = 0.52) as reference measures. Cut points approximated 1.0 for EMA fatigue intensity and approximated 2.0 for EMA fatigue interference when using either reference measures.

### Distribution-based scores

Across the 7-day assessment period, the between-person reliability ranged from 0.54 to 0.93, The between-person reliability of EMA fatigue exceeded 0.80 after 3 days of assessment (fatigue intensity = 0.81; fatigue interference = 0.84) and 0.90 after 6 days of assessment (fatigue intensity = 0.91; fatigue interference = 0.92). For EMA fatigue intensity, the smallest amount of change estimated using the SEM ranged from 1.19 to 1.56 and the largest amount of change estimated using pre-treatment SD ranged from 1.50 to 1.93. For EMA fatigue interference, the smallest amount of change estimated using the SEM ranged from 1.30 to 1.64 and the largest amount of change estimated using pre-treatment SD ranged from 1.66 to 2.11, Table 4.

**Table 4:** Distribution-based indicators of MCID for EMA fatigue intensity and fatigue interference

Measure	Days	r	ICC	SD	½SD	SEM	Cohen's *d*
Fatigue Intensity	1 Day	0.54	0.34	1.93	0.96	1.56	0.41
	2 Days	0.73	0.33	1.63	0.81	1.33	0.46
	3 Days	0.81	0.33	1.55	0.77	1.27	0.54
	4 Days	0.86	0.34	1.51	0.76	1.22	0.56
	5 Days	0.89	0.36	1.51	0.75	1.21	0.57
	6 Days	0.91	0.36	1.50	0.75	1.20	0.57
	7 Days	0.93	0.37	1.50	0.75	1.19	0.59
Fatigue Interference	1 Day	0.60	0.40	2.11	1.06	1.64	0.32
	2 Days	0.76	0.36	1.78	0.89	1.42	0.41
	3 Days	0.84	0.38	1.76	0.88	1.39	0.46
	4 Days	0.88	0.38	1.70	0.85	1.34	0.50
	5 Days	0.90	0.38	1.68	0.84	1.32	0.51
	6 Days	0.92	0.38	1.66	0.83	1.31	0.52
	7 Days	0.93	0.39	1.66	0.83	1.30	0.53

*ICC* Intra-Class Correlation, *r* Between-persons Reliability of Average number of days, *SD* Standard Deviation of pre-treatment (baseline) scores, *SEM* Standard Error of Measurement = SD_baseline_(√1 − ICC)

After 7 days of assessment, Cohen’s d estimates were the strongest for both EMA fatigue intensity (0.59) and EMA fatigue interference (0.53), and distribution-based indices of variability (SEM and ½ pre-treatment SD) for the EMA fatigue measures approximated 1.0, Table 4.

### Anchor- and distribution-based scores

On combining the distribution-based measures with each of the two anchor-based measures, compared to the PGIC, the accuracy estimates of the distribution-based EMA fatigue measures with the MFIS as the anchor were slightly higher and above 0.60. The accuracy for fatigue intensity = 0.68 and fatigue interference = 0.68 and the corresponding Youden’s index for fatigue intensity = 0.08 and fatigue interference = 0.09 were the highest for pre-treatment ½SD for EMA fatigue intensity = 0.75 and EMA fatigue interference = 0.83 when using MFIS as the anchor, see Table 5.

**Table 5:** Combining anchor- and distribution-based methods

Measure	Distribution-based indicators	Accuracy	Sensitivity	Specificity	Youden's Index
PGIC: moderately better vs almost the same or less					
Fatigue intensity	1SD = 1.50	0.45	0.97	0.07	0.04
	½SD = 0.75	0.47	0.92	0.13	0.06
	SEM = 1.19	0.45	0.95	0.08	0.03
Fatigue interference	1SD = 1.66	0.46	0.97	0.09	0.06
	½SD = 0.83	0.46	0.91	0.12	0.03
	SEM = 1.30	0.46	0.95	0.09	0.04
MFIS: ≥ 10 points vs < 10 points					
Fatigue intensity	1SD = 1.50	0.68	0.98	0.06	0.04
	½SD = 0.75	0.68	0.95	0.13	0.08
	SEM = 1.19	0.67	0.97	0.07	0.04
Fatigue interference	1SD = 1.66	0.68	0.98	0.05	0.02
	½SD = 0.83	0.68	0.95	0.14	0.09
	SEM = 1.30	0.68	0.97	0.10	0.07

Accuracy = (TP + TN)/Total

Sensitivity = TP/(TP + FN)

Specificity = TN/(TN + FP)

*SD* Standard Deviation of pre-treatment (baseline) scores, *SEM* Standard Error of Measurement = SD_baseline_(√1 − r)

Youden's Index = sensitivity + specificity—1.00. Value ranges from − 1 through 1, 0 indicates the proportion of true positives and true negatives are equal, i.e. the test is useless. 1 indicates that there are no false positives or false negatives, i.e. the test is perfect. The index gives equal weight to false positive and false negative values, so all tests with the same value of the index give the same proportion of total misclassified results

## Discussion

To our knowledge, this is the first study to determine MCIDs in EMA assessed fatigue outcomes in individuals with MS. Following recommendations suggesting use of multiple anchors and combining both anchor- and distribution-based methods [45–47], we found that a difference ranging from 0.75 to 1.30 represents a minimal and clinically important difference in EMA fatigue intensity and fatigue interference on the 0–10 NRS. These MCIDs are critical to interpreting findings of future clinical trials evaluating fatigue outcomes via EMA and contribute to quality of life in pwMS by providing a more precise, ecological, and meaningful measure of change, either through targeted interventions or due to disease progression.

There are several advantages to our study. The MCIDs and effects size estimates for the EMA fatigue outcomes in this study fall within the range of MCIDs (effect sizes) for improvement in fatigue intensity on the 0–10 NRS that range from 0.30 to 1.12 (0.4–0.8) [48–50], and on the 0–100 NRS that range from 1.4 to 13.9 (0.3–0.6) [51–56] in studies that have used other patient reported outcome measures of fatigue. Considering the high variability experienced with fatigue and its inadequate assessment using retrospective surveys as seen in prior studies, this study used pre-to-post treatment mean change fatigue scores based on multiple daily real-time assessments of fatigue, which provides ecological validity. In addition, we used multiple EMA measures that included mental and physical fatigue intensity and fatigue interference for a more comprehensive assessment of fatigue. Reliability assessments of our estimates showed that the average between-person reliability for both the EMA fatigue intensity and EMA fatigue interference increased across the assessment period of increasing duration (day 1 through days 7). A minimum of six days were needed to attain the highest reliability ≥ 0.90; with values exceeding 0.80 just after three days and exceeding 0.70 just after two days. Similarly, the highest ICCs that corresponded to the smallest SEM and SD values in EMA fatigue intensity = 1.19 and EMA fatigue interference = 1.30 at baseline were after six to seven days. Thus, suggesting that longer protocols are needed to detect subtle intraindividual changes in fatigue. While the degree of improvement in EMA fatigue outcomes may vary considerably across unmeasured subject specific characteristics, the within- and between-persons mean change scores in EMA fatigue intensity and interference were not different by treatment arm and demographic characteristics in this study.

Our study is not without limitations. First, the PGIC is a generic and asymmetric scale applicable to a wide variety of conditions and treatments lacking specificity to change in fatigue and assessments at both ends of the spectrum (improvement – worse), and its utility is therefore diluted by the lived experience of other chronic symptoms and overall functional status. Use of domain-specific transition questions are shown to have higher construct validity as anchors for determining clinically important differences in health measures focused on a single domain than either global disease or general health transition questions [57]. Though we used a generic and asymmetric PGIC scale, individuals who reported no change or reported getting worse on the PGIC showed no increase in their EMA fatigue intensity scores (within-persons change score = − 0.10) and only a slight increase in their EMA fatigue interference scores (within-person change score = 0.37) over the 12-week post treatment period. Second, both anchor measures are prone to recall bias and are unlikely to reflect an individual’s momentary assessment of fatigue.

Third, while the NRS for fatigue intensity has been validated and used in other disease conditions [58, 59], and the composite measures in this study demonstrate convergent validity with the MFIS, further studies are needed to demonstrate validity of the EMA mental and physical fatigue items and the composite fatigue measures in pwMS. Fourth, while the correlations between the anchor-based and EMA measures in our study are low and likely due to the above stated limitations, they fall within the recommended minimum correlation of at least 0.3–0.35 between the change score and the anchor measure [46] and what has been reported for traditional fatigue measures and other constructs found in prior studies [48–56]. Fifth, given that the MDC was greater than the SEMc for each of the fatigue measures which likely accounts for change beyond random variation, minimal changes not due to random variation do not provide an individual’s perspective of clinically important change despite being sample specific, i.e. even with large sample sizes and wide distributions, the estimated MDC may not reflect actual change. Therefore, given the above stated limitation with the MDC we decided to combine two approaches to determine the best estimate of the MCID in EMA fatigue and find that our estimates of MCID for EMA fatigue are robust measures of change assessed against two approaches using a patient reported global outcome of change and a specific outcome of fatigue impact and distribution-based estimates not influenced by treatment. Sixth, due to the potential for overmedicalization of naturally fluctuating symptoms, we recommend that clinical settings using EMA should aim to thoughtfully educate patients on the natural variation of the symptoms and carefully customize treatments based on the fluctuations and the severity of the symptoms to enhance compliance and effectiveness of the treatment. Lastly, our MCID estimates for EMA fatigue intensity and interference may not be applicable to select subgroups not adequately represented in this RCT, that said, our RCT sample included all adults with MS (all subtypes), regardless of age, disease duration, disability level, or disease modifying therapy status, and is representative of the broader population of pwMS, who are mostly female, white, and between ages 35–64 years old [60].

## Conclusions

This study is the first to contribute important information on meaningful changes in EMA measures of fatigue, enabling effective use of EMA to aid in sample size calculations to plan adequately powered clinical trials and assess fatigue treatment outcomes in a patient-centered manner.

## Data Availability

Not applicable.
